# How promising are HIV-1-based virus-like particles for medical applications

**DOI:** 10.3389/fcimb.2022.997875

**Published:** 2022-10-07

**Authors:** Sofia A. Martins, Joana Santos, Rúben D. M. Silva, Cátia Rosa, Sandra Cabo Verde, João D. G. Correia, Rita Melo

**Affiliations:** ^1^ Centro de Ciências e Tecnologias Nucleares, Instituto Superior Técnico, Universidade de Lisboa, Lisboa, Portugal; ^2^ Departamento de Engenharia e Ciências Nucleares, Instituto Superior Técnico, Universidade de Lisboa, Lisboa, Portugal

**Keywords:** virus-like particles, HIV-1-based virus-like particles, medical applications, vaccines, drug delivery

## Abstract

New approaches aimed at identifying patient-specific drug targets and addressing unmet clinical needs in the framework of precision medicine are a strong motivation for researchers worldwide. As scientists learn more about proteins that drive known diseases, they are better able to design promising therapeutic approaches to target those proteins. The field of nanotechnology has been extensively explored in the past years, and nanoparticles (NPs) have emerged as promising systems for target-specific delivery of drugs. Virus-like particles (VLPs) arise as auspicious NPs due to their intrinsic properties. The lack of viral genetic material and the inability to replicate, together with tropism conservation and antigenicity characteristic of the native virus prompted extensive interest in their use as vaccines or as delivery systems for therapeutic and/or imaging agents. Owing to its simplicity and non-complex structure, one of the viruses currently under study for the construction of VLPs is the human immunodeficiency virus type 1 (HIV-1). Typically, HIV-1-based VLPs are used for antibody discovery, vaccines, diagnostic reagent development and protein-based assays. This review will be centered on the use of HIV-1-based VLPs and their potential biomedical applications.

## 1 Introduction

Nanotechnology is defined as a fusion of state-of-the-art science and engineering where the production or assembly is aimed at the nanometer scale (1-100 nm) (Chenthamara et al., 2019). Regarding biomedical applications and drug development, it has emerged as a field focused on overcoming the limitations of conventional drug delivery (Mitchell et al., 2021). NPs can boost the transport of the desired cargo across cell membranes, enhance the stability and solubility of packaged drugs, and lengthen circulation times (Mitchell et al., 2021). However, despite the potential exhibited by NPs in general, only a scarce number of lipid and protein-based formulations have been authorized in the clinical setting (Dinndorf et al., 2007; Bovier, 2008; Wei et al., 2008; Barenholz, 2012; Mullard, 2018).

VLPs may be an attractive alternative for drug delivery, since they take structural cues from viruses, which are natural nanoscale delivery vehicles, and therefore are able to circumvent some of the limitations associated with other NPs (Rohovie et al., 2017; Benjamin et al., 2020). The size of VLPs varies from 20 to 200 nm and their biological nature confers them several characteristics that are particularly valuable for medical purposes: easy to manufacture, capable of self-assembling, modifiable, stability, biocompatibility, immunogenicity, and ability to cross cellular membranes and deposit the desired cargo at the cytoplasm (Manolova et al., 2008; Lee et al., 2009; Zeltins, 2013; Mohsen et al., 2017; Benjamin et al., 2020). Several studies have reported that VLPs are versatile platforms that can be used as immunogens but also as delivery vehicles for therapeutic cargos and for imaging agents (Flexman et al., 2008; Peabody et al., 2008; Kaczmarczyk et al., 2011; Kelly et al., 2015; Thong et al., 2019; Francica et al., 2021). Nonetheless, there are still some drawbacks that need to be taken into consideration (Rohovie et al., 2017). While small size VLPs (< 30 nm diameter) can be internalized, larger VLPs may be taken up by phagocytic cells, which in turn may originate inflammation (Tran et al., 2017). VLPs can also induce strong immune responses owing to the structural pattern of capsid proteins that is analogous to the native virus (Donaldson et al., 2018). These issues can be addressed *via* modification of the VLP surface (Rohovie et al., 2017). VLPs can be constructed in the laboratory through heterologous expression in different platforms including prokaryotic cells, yeast, insect cells, plant cells and mammalian platforms (Nooraei et al., 2021). Most VLPs are constructed with proteins from an individual virus, but proteins derived from different viruses can be used to generate chimeric VLPs (Nooraei et al., 2021).

These nanostructures can be categorized in different classes (Manolova et al., 2008; Lee et al., 2009; Mohsen et al., 2017). Regarding the presence or absence of a lipid envelope, VLPs can be classified as enveloped or non-enveloped, respectively (Chroboczek et al., 2014; Zdanowicz and Chroboczek, 2016). Enveloped VLPs acquire their lipid membrane from their expression hosts, during assembly and budding, whereas non-enveloped VLPs are composed of one or more structural viral proteins and can thus be further divided in single- or multiple-capsid protein VLPs (Nooraei et al., 2021). Single-capsid protein VLPs display the simplest structure and can therefore be assembled in both prokaryotic and eukaryotic expression platforms, as well as in cell-free systems (Kirnbauer et al., 1992; White et al., 1997; Chen et al., 2001; Bundy et al., 2008). Some examples of single-capsid protein VLPs include HPV VLPs and Norwalk VLPs (Kirnbauer et al., 1992; White et al., 1997). Multi-capsid protein VLPs are more complex and thus require higher eukaryotic platforms (Crawford et al., 1994; Rodriguez-Limas et al., 2011; Kurokawa et al., 2021). Rotavirus VLPs are among multi-capsid protein VLPs (Crawford et al., 1994). Enveloped VLPs possess a lipid bilayer derived from the expression host in which glycoprotein spikes can be embedded and therefore prompt the production of neutralizing antibodies (Lua et al., 2014). These VLPs are structurally more complex and more environmentally sensitive than non-enveloped VLPs, and thus require eukaryotic expression systems (Warfield et al., 2003; Cervera et al., 2013; Thompson et al., 2015). Their integrity and stability can be compromised by purification processes, sample handling and temperature variations, which can lead to a reduction in immunogenicity (Nooraei et al., 2021). Enveloped VLPs comprise Influenza VLPs, HIV VLPs and Ebola VLPs, among others (Warfield et al., 2003; Cervera et al., 2013; Thompson et al., 2015).

VLPs can induce strong humoral and cellular immune responses due to their dense repetitive structure (Wagner et al., 1998; Chackerian et al., 1999; Villa et al., 2006). This structural arrangement can stimulate B cell activity and increase antibody yield (Wagner et al., 1998). The uptake of VLPs and consequent degradation by antigen-presenting cells leads to T cell activation. These features, together with the lack of viral genome, which hinders replication within the target cell, make VLPs relevant vaccine candidates.

Owing to the structural properties of VLPs, they can be engineered to encapsulate proteins, peptides, nucleic acids, imaging agents, drugs, quantum dots or other types of nanoparticles (Li et al., 2009; Ashley et al., 2011; Kaczmarczyk et al., 2011). On the outer surface they can also display targeting ligands that will make the VLP specific to a cell, tissue, or organ, changing the natural tropism of the VLP (Patel and Swartz, 2011; Pokorski et al., 2011). Surface modification of VLPs can be accomplished by either chemical techniques or genetic modifications (Bustos-Jaimes et al., 2017; Zackova Suchanova et al., 2017). Non-enveloped VLPs can be modified through a covalent strategy that harnesses amino acid residues on the surface (Zhao et al., 2011; Galaway and Stockley, 2013). Natural amino acids can be used, although their location may not satisfy requirements regarding reactivity or surface accessibility (Stephanopoulos et al., 2010). It is possible to circumvent these limitations by genetically introducing or removing amino acids at pertinent locations (Stephanopoulos et al., 2010). Alternatively, viral capsid proteins can be fused with target proteins and peptides and therefore decorate non-enveloped VLPs internally or externally (Middelberg et al., 2011; Pokorski et al., 2011). This strategy permits the direct linkage of the desired targeting moiety to the viral capsid proteins but is limited to proteins and peptides (Pokorski et al., 2011). Moreover, these peptides must be smaller in size in order not to alter VLP function (Smith et al., 2013). Lastly, non-enveloped VLPs can be non-covalently modified through electrostatic (Kostiainen et al., 2011), protein-protein (Patterson et al., 2012), protein-nucleotide (Galaway and Stockley, 2013), and protein-metal (Uchida et al., 2012) interactions. Non-covalent modifications are normally reversible, which may be a disadvantage when long-term stability is required (Smith et al., 2013). Enveloped VLPs, on the other hand, are more adequate for the presentation of membrane proteins, given the origin of their envelope (Grgacic and Anderson, 2006). This type of VLP can also be chemically or genetically modified, the latter occurring through the fusion of a protein of interest with a full-length viral protein or a transmembrane domain (Pan et al., 2010; Wei et al., 2011; Carvalho et al., 2016). Notwithstanding, the correct folding and function of proteins, and the host’s ability to incorporate the proteins of interest in the VLP must be taken into account when functionalizing enveloped VLPs (Grgacic and Anderson, 2006). The ability to modify VLPs thus opens novel avenues for the development of targeted delivery.

The structural proteins from viruses such as human papillomavirus (HPV), hepatitis B virus (HBV) and human immunodeficiency virus (HIV) are typically employed for the production of VLPs in research (Nooraei et al., 2021). Several VLP-based vaccines have been approved by the FDA, namely vaccines against HPV (Gardasil ^®^ and Cervarix™) (Einstein et al., 2009), Hepatitis B (Engerix-B^®^ and Recombivax^®^) (Lacson et al., 2005), Hepatitis E (Hecolin^®^) (Zhang et al., 2014) and malaria (Mosquirix™) (Laurens, 2020). VLP-based vaccines against infectious agents, including Influenza virus (Fries et al., 2013) and SARS-CoV-2 (Lu et al., 2020) are currently under development.

HIV-1-based VLPs require just one polyprotein to self-assemble, making them particularly attractive nanoplatforms (Cervera et al., 2019). This type of VLPs has been mainly studied as a vaccine given its ability to boost strong immune reactions (Cervera et al., 2019). One of the most challenging viruses in the field of vaccination is in fact HIV, given the lack of efficient preventive strategies for the syndrome caused by this virus, and HIV-1-based VLPs could be harnessed as platforms for HIV vaccination (Weber et al., 1995; Tagliamonte et al., 2010; Franco et al., 2011; Ao et al., 2019; Ao et al., 2021; Liu et al., 2021; Beltran-Pavez et al., 2022). The only VLP-based HIV-1 candidate in phase I/II trials was the p17/p24:ty VLP (Weber et al., 1995) (Peters et al., 1997). This vaccine candidate did not induce significant humoral and cellular immune responses (Weber et al., 1995) (Peters et al., 1997). Several assays have also been conducted to test the potential of these VLPs as vaccine candidates for other viruses, namely Dengue, Influenza, Foot-and-Mouth Disease and SARS-CoV-2 (Steel et al., 2010; Chua et al., 2013; Venereo-Sanchez et al., 2016; Fontana et al., 2021; Boix-Besora et al., 2022). The main difficulty of using HIV-based VLPs as vaccines is the selection of adequate immunogens to elicit a significant immune response (Cervera et al., 2019).

Although HIV-1-based VLPs have been mainly explored as vaccination nanoplatforms, they may be harnessed for other applications. The packaging of active molecules as well as the surface modification for targeting bestow VLPs with the ability to function as delivery platforms (Zdanowicz and Chroboczek, 2016). VLPs can evade the degradation route followed by conventional NPs, and tackle the affinity of the native virus for specific tissues to achieve specificity, reducing off target effects and sustaining drug activity and stability (Zdanowicz and Chroboczek, 2016). HIV-based VLPs have thus been recently exploited as nanocarriers for the delivery of a broad range of molecules, attaining promising results that need to be further validated to establish VLPs as delivery vessels in the medical field (Robert et al., 2017; Lyu et al., 2019; Indikova and Indik, 2020; Hamilton et al., 2021).

This review compiles an updated set of HIV-1-based VLPs, addressing their current applications as viral vaccines, namely those with anticancer properties, as well as tools for targeted therapy. As biomedical application of VLPs is considered a hot topic, there are a few reviews on this subject (Zeltins, 2013; Zdanowicz and Chroboczek, 2016; Rohovie et al., 2017; Benjamin et al., 2020; Nooraei et al., 2021). However, as far as we know, there are no reviews that tackle the state of the art of work developed with HIV-1-based VLPs for medical applications.

## 2 HIV-1-based VLPs

Within the variety of viral particles under study, HIV-1-based VLPs have gained importance due to their recognized applicability as vaccines (Weber et al., 1995; Tagliamonte et al., 2010; Franco et al., 2011; Ao et al., 2019; Ao et al., 2021; Liu et al., 2021; Beltran-Pavez et al., 2022). HIV contains, among other components, the Gag polyprotein, which can self-assemble into VLPs without requiring any other viral protein, making this type of VLP more attractive (Bednarska et al., 2020).

HIV-1 is the most common subtype of HIV, a virus that derives from primates and targets the human immune system, giving rise to the acquired immunodeficiency syndrome (AIDS) (Campbell and Hope, 2008). Given that HIV-1 belongs to the group of enveloped retroviruses, its infection process begins with the contact of the viral envelope proteins with proteins on the surface of macrophages and T lymphocytes (Campbell and Hope, 2008). This contact elicits the merging of viral and host membranes (Campbell and Hope, 2008). The viral components are transported to the cell cytoplasm, and the viral nucleoprotein complex transits to the nucleus, leading to the reverse transcription of the viral genetic material into DNA and culminating in its incorporation into the host genome (Campbell and Hope, 2008). This also permits the expression of structural and accessory proteins that are required for assembly of viral particles that have the ability to infect other cells (Campbell and Hope, 2008).

**Figure 1:**
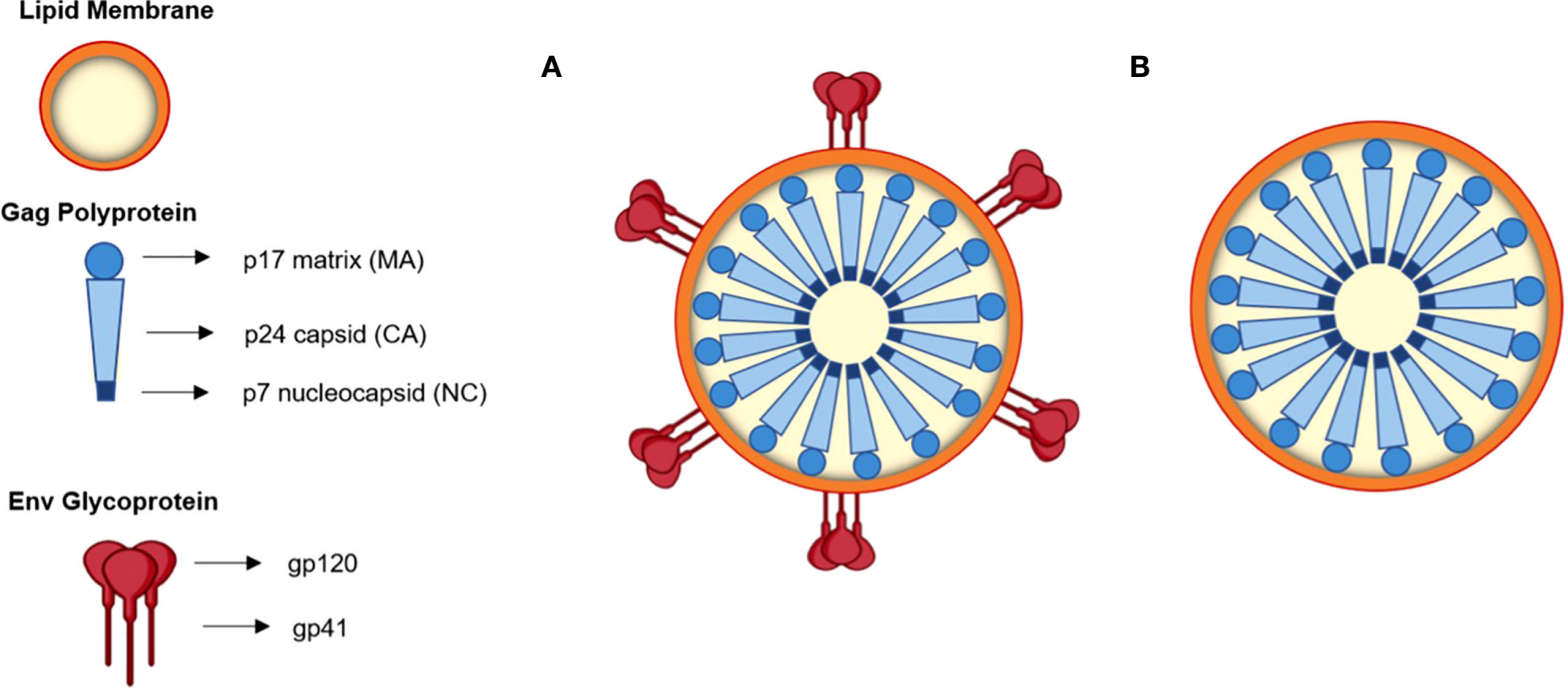
Schematic representation of an HIV-1 based VLP **(A)** and an HIV-1 Gag VLP **(B)**.

The primary proteins of replicative retroviruses are encoded in the HIV-1 RNA genome (9.7 kb) (Freed, 2001; Cervera et al., 2019). Apart from these elements, the three main genes encoded in the HIV-1 genome are *gag* (group-specific antigen), *pol* (polymerase) and *env* (envelope glycoprotein) (Freed, 2001; Cervera et al., 2019). The *gag* gene encodes Pr55^Gag^, a polyprotein precursor that undergoes cleavage by the viral protease (PR), originating the proteins matrix (MA or p17), capsid (CA or p24), nucleocapsid (NC or p7) and p6 (**Figure 1**), as well as two small spacer peptides, p2 and p1 (Freed, 2001; Cervera et al., 2019). The *pol* gene originates Pr160^GagPol^, a large polyprotein precursor, which is also cleaved by PR to generate distinct *pol*-encoded enzymes, PR, reverse transcriptase (RT), and integrase (IN) (Freed, 2001; Cervera et al., 2019).

Two envelope glycoproteins (Env), the surface Env glycoprotein gp120 and the transmembrane glycoprotein gp41 are formed upon processing of the polyprotein precursor gp160 by a cellular protease (Freed, 2001; Cervera et al., 2019). gp41 is a transmembrane glycoprotein that mediates the fusion of membranes and prompts viral core entry, whereas gp120 is an outer layer glycoprotein responsible for viral infection, facilitating HIV entry into the host cell. The latter displays affinity to the CD4 receptor and CCR5 and CXCR4 co-receptors of the cell (Freed, 2001; Cervera et al., 2019). Both proteins form heterotrimeric complexes in the lipid bilayer of budding virions (Freed, 2001; Cervera et al., 2019).

The HIV-1 genome also encodes numerous regulatory and accessory proteins, namely Tat, which is necessary for transcription from the HIV-1 long terminal repeat, and Rev, which is involved in the transit of viral RNAs to the cytoplasm (Freed, 2001). Other proteins include Vpu, Vif, Vpr and Nef (Freed, 2001; Cervera et al., 2019).

HIV-1-based VLPs are robust nanoplatforms of around 150 nm in size (**Figure 1A**) that comprise structural internal polyproteins, a lipidic envelope derived from the host membrane and glycoproteins (González-Domínguez et al., 2016; Cervera et al., 2019). The stability and the structure of these VLPs, together with the ability to be functionalized, make them promising recombinant vaccines and delivery vessels (Cervera et al., 2019).

Two types of HIV-1-based VLPs can be formed. If the host cell expresses only the *gag* gene, a Gag-VLP (**Figure 1B**), that resembles immature HIV virions, will be produced (Fernandes et al., 2020). If the *env* gene is co-expressed with *gag*, then a Gag-Env VLP (**Figure 1A**) will be assembled and these enveloped proteins will be present on the VLP surface (Zhang et al., 2021). The primary advantages of HIV-1-based VLPs over other VLPs include the ability to self-assemble with just one polyprotein (Cervera et al., 2013), to display HIV-1 epitopes on the surface and thus elicit innate and adaptive immune responses as they are cleared into the lymph nodes and are subsequently taken up by antigen-presenting cells (APCs) (Gonelli et al., 2019). Another important advantage is related to the fact that they are enveloped, which may be relevant for the presentation of membrane proteins (Boix-Besora et al., 2022). Despite that, these VLPs also have some limitations (Gonelli et al., 2019). Several HIV-1-based VLPs have been constructed with Gag-only expression systems, which allow for the formation of VLPs that resemble immature virions (Buonaguro et al., 2001; Valley-Omar et al., 2011). This occurs due to the lack of PR, which is responsible for the proteolytic cleavage of Gag and consequent formation of mature virions (Freed, 2001). In the absence of this enzyme, it is possible to achieve high yields of HIV-1 VLPs with incorporation of Env into the lipid membrane (Chojnacki et al., 2012). Nonetheless, the structure of immature virions hampers the clustering and lateral movement of Env on the lipid membrane, leading to a sparse distribution of Env spikes (Chojnacki et al., 2012). This could dwindle the antibody response. Conversely, HIV-1-based VLPs that mimic mature virions may be able to integrate their viral genetic material into the host genome (Alvarez-Fernandez et al., 2012). This may lead to the need to inactivate the VLPs, which further complicates the production process (Gonelli et al., 2019). Additionally, VLPs expressed *in vitro* need to be isolated from exosomes and microvesicles that are present in the cell supernatant, which may hinder large scale production (Steppert et al., 2017). Moreover, host cell proteins may be incorporated into HIV-1 VLPs, and these may persist following purification (Gonelli et al., 2019). Cross-species immune responses may thus occur, hampering immunogenicity studies (Gonelli et al., 2019). Strategies concerning the vector used for expression could overcome this limitation (Gonelli et al., 2019).

### 2.1 HIV-1-based VLP assembly and budding process

As aforementioned, the Gag polyprotein can self-assemble into a VLP without requiring other proteins and viral genetic material (Perlman and Resh, 2006; Gutiérrez-Granados et al., 2013; González-Dominguez et al., 2020). The different domains of the Gag protein must interact in a coordinated manner for assembly to occur. For instance, VLP formation and the attachment of the Gag precursor to the internal layer of the cell membrane both depend on the N-terminal glycine residue from p17 MA (site of myristic acid attachment) (Deml et al., 2005; Lavado-Garcia et al., 2021). p17 MA also intervenes in the integration of the Env glycoprotein into the VLP surface. Further studies also suggest that the p7 domain is necessary to direct and attach Gag to the cellular membrane (Deml et al., 2005).

The PR enzyme allows the HIV-1 based VLP to move laterally and clustering of the Env proteins on the VLP surface (Gonelli et al., 2019).

The Gag polyproteins are expressed in the cell cytoplasm, in cytosolic ribosomes (Deml et al., 2005) and are transported to the plasma membrane through the endosomal sorting complex. These complexes also coordinate the assembly and oligomerization of the Gag and the subsequent budding process, in which VLPs acquire a lipid envelope from the host cell (Deml et al., 2005; Gutiérrez-Granados et al., 2013; González-Domínguez et al., 2016; Lavado-García et al., 2021). Gag binding to the plasma membrane is carried out through the p17 matrix domain (Tedbury and Freed, 2014) with buds being released later from the cell membrane to form Gag-VLPs (Deml et al., 2005; Tedbury and Freed, 2014; Gonelli et al., 2019).

HIV Gag also encapsulates two copies of the viral genetic material that possess a HIV packaging signal dubbed the ψ-site during assembly (Aldovini and Young, 1990). Mutations in this site thwart the incorporation of viral genomic RNA, but do not prevent the encapsulation of host cell derived RNA (Aldovini and Young, 1990; Muriaux et al., 2002). This occurs due to RNA binding to residues that are present in different domains of Gag, namely the NC domain, and mutations in these domains hamper the packaging of RNA and the assembly of viral particles (Alfadhli et al., 2005; Rein et al., 2011). It has therefore been depicted that RNA functions as a scaffold for Gag’s ability to assemble viral particles, with nucleotides as short as 20-40 bases being able to support assembly *in vitro* (Cimarelli et al., 2000). It seems that Gag molecules, when bound to nucleic acids, interact with other nucleic acid-bound Gag molecules, and this results in VLP assembly, indicating that oligomerization plays a relevant role in this process (Ma and Vogt, 2002). The production of HIV Gag VLPs in expression systems such as bacteria, yeast, insect cells and mammalian cells may thus lead to the encapsulation of host cellular RNA, which is highly undesirable for the development of vaccines (Valley-Omar et al., 2011). This is one of the main hindrances of the *in vitro* production of HIV-1-based VLPs for therapeutic applications. However, it has been reported that chimeric Gag proteins in which the NC domain was replaced with extrinsic sequences are capable of assembling and forming VLPs at comparable levels to wild-type (WT) Gag proteins (Bennett et al., 1993; Zhang et al., 1998). Zhang et al. (Zhang et al., 1998) replaced NC with polypeptides that are structurally well-characterized and assessed particle assembly. The resulting chimeras allowed to ascertain the mechanism through which NC intervenes in assembly (Zhang et al., 1998). It was established that NC may form interprotein interactions, and that its replacement with protein domains that play a similar role prompt VLP assembly without significant viral RNA encapsidation (Zhang et al., 1998). Although not established, this study shows that protein engineering may be a promising strategy to prevent the encapsulation of host cell derived RNA in VLPs.

If the host cell expresses not only the *gag*, but also the *env* gene, then Env-Gag VLPs will be assembled (Zhang et al., 2021). The Env glycoprotein is translated in the endoplasmic reticulum and migrates *via* the Golgi complex to the cell membrane (Cervera et al., 2019).

There are four models that explain the incorporation of the enveloped glycoproteins into the Gag-VLP (Tedbury and Freed, 2014). The first model is based on a passive mechanism in which the Env proteins present on the assembly site in the plasma membrane will be incorporated into the VLP surface as the budding occurs (Tedbury and Freed, 2014). The second model is related to the co-targeting of Gag and Env, where packaging of the Env glycoproteins is enhanced by directing Gag and Env to lipid rafts in the plasma membrane (Tedbury and Freed, 2014). The other models implicate specific protein-protein interactions, which can be direct or indirect. The direct Gag-Env interaction model suggests that a direct interaction between the MA of the Gag and the cytoplasmic tail (CT) of the gp41 during particle assembly is necessary for Env incorporation into the VLP (Tedbury and Freed, 2014). The indirect mode, on the other hand, proposes the presence of a cellular factor that binds to Env and Gag, bridging their interaction. This last model suggests that the Env incorporation is cell type-dependent (Tedbury and Freed, 2014).

### 2.2 Major developments on HIV-1-based VLP production

The production of HIV-1-based VLPs is performed by transfection methods of a specific cell type. Different eukaryotic expression platforms, such as mammalian and insect cells have been studied. The selection of the expression system to be used determines the production yield, as well as the glycosylation profile (Gutiérrez-Granados et al., 2016; Fuenmayor et al., 2018a; Fernandes et al., 2020; Puente-Massaguer et al., 2021; Lavado-Garcia et al., 2022). The correct glycosylation pattern is crucial for the stability, solubility, safety, and efficacy of biopharmaceutical products (Gupta and Shukla, 2018). In addition, the glycosylation profile of VLPs affects the downstream processing, as it determines which separation and purification strategies are required to eliminate inadequate glycoforms (Lavado-Garcia et al., 2022). Several researchers work on different methods to optimize the transfection process to increase yields, reduce production costs and create scalable methods in producing structurally correct and immunogenic HIV-1 based-VLPs (Cervera et al., 2019).

The most extensively explored mammalian expression systems to produce these enveloped VLPs are the human embryonic kidney 293 (HEK-293) cells. This cell line is used in both research and industrial environments as it is easy to handle (Ausubel et al., 2012; Dietmair et al., 2012; Fuenmayor et al., 2018a; Hengelbrock et al., 2022). Mammalian cell lines display a lower productivity but are the most adequate expression platforms given their ability to confer complex post-translational modifications (Cervera et al., 2019). Production levels of up to 2.7 x 10^9^ VLPs/ml have been achieved through transient transfection of HEK-293 cells (Cervera et al., 2013). Strategies like extended gene expression and the incorporation of chemical additives can significantly improve the production yield to up to 15.6-fold (Fuenmayor et al., 2018b). It is thus possible to attain adequate production levels through the combination of different methods. Regarding glycosylation, it has been reported that HIV-1 Gag VLPs produced in this cell line display a glycosylation pattern composed of structures of high mannose, hybrid and complex glycans (Lavado-Garcia et al., 2022). High mannose structures elicit lower antibody titres when in comparison with complex glycans (Lin et al., 2013). This might occur due to a masking effect provoked by glycans in viral particles, which prevents detection by the immune system and handicaps the development of effective vaccines (Lee et al., 2016; Casalino et al., 2020). Notwithstanding, hybrid and complex glycans can induce a more robust immune reaction (Lin et al., 2013). The presence of sialic acid in the *N-* and *O-*glycans of HEK-293-derived VLPs may also encourage macrophage recognition and consequent presentation to APCs (Jobe et al., 2016; Lavado-Garcia et al., 2022). It is important to note that upon transfection of HEK-293 cells, the formation of small extracellular vesicles (EVs) was reported, and this can affect glycan density and bioprocessing (Lavado-Garcia et al., 2022). Moreover, the HEK-293 cell line can confer other modifications to the VLP like *N‐*myristoylation, which is responsible for the transit of Gag molecules to the cell membrane for budding (Lavado-Garcia et al., 2021). However, the overexpression of *N‐*myristoylation can hinder Gag assembly due to an imbalance in the myristoyl switch or due to the *N-*myristoylation of different molecules, which can block the Gag binding sites and subsequently inhibit VLP assembly (Lavado-Garcia et al., 2021). These modifications influence the development of purification strategies and must therefore be taken into consideration during downstream processing, with the aim of improving VLP production technology (Lavado-Garcia et al., 2022).

The CAP-T (CEVEC Pharmaceuticals) cell line is also employed in the manufacture of HIV-1-based VLPs (Gutiérrez-Granados et al., 2016; Gutiérrez-Granados et al., 2017). These cells grow in a serum-free medium and suspension, while having high cell densities and expressing recombinant proteins with human post-translational modifications (Gutiérrez-Granados et al., 2016; Gutiérrez-Granados et al., 2017). Following optimization of transient transfection, a concentration of 5.8 x 10^10^ VLPs/ml was achieved, and the obtained VLPs exhibited a similar morphology to those produced in HEK-293 (Gutiérrez-Granados et al., 2016). Such features give CAP-T the ability for large-scale transient transfection, which is a huge advantage when moving from the development to the production stage (Gutiérrez-Granados et al., 2016; Gutiérrez-Granados et al., 2017). Albeit promising, further studies are required to ascertain the ability of this cell line to produce adequately modified HIV-1-based VLPs with the potential to be employed in the medical setting.

Insect cell lines are characterized by producing high titers and being simple to scale up and are therefore frequently used in vaccine production (Cervera et al., 2019). They can also confer several post-translational modifications that are analogous to those performed by mammalian cells (Cervera et al., 2019). The main limitation of this type of expression system is the incorporation of glycans that are significantly different from human glycans, which hampers the immunological features of the produced VLPs (Lancaster et al., 2016). Extrinsic glycan signatures derived from nonhuman host platforms may provoke adverse effects like allergies or treatment rejection (Md et al., 2021). There are several factors that affect the production of HIV VLPs in insect cell lines with the baculovirus vector expression system, including multiplicity of infection (MOI), cell line, cell density and time of infection (Cervera et al., 2019). These parameters were assessed in the development of HIV-1-based VLPs in two cell lines*, Trichoplusia ni Pro™* (*High Five*) and *Spodoptera frugiperda* (*Sf9*) (Pillay et al., 2009). Higher yields of HIV-1-based VLPs of around 400 ng/ml were obtained in the *High Five* cell line, at a cell density of 1 x 10^6^ cells/ml and an infection time of 96 h post infection (Pillay et al., 2009). The main shortcoming of employing baculovirus expression systems is the unfeasibility of removing these baculoviruses from the final product, which will greatly impact the function of the produced VLPs (Cervera et al., 2019). Accordingly, the development of stable insect cell lines is required (Cervera et al., 2019). HIV-1 VLPs were successfully produced in *High Five* cells through constitutive expression of the Gag polyprotein (Tagliamonte et al., 2010). This was also achieved in the *Sf9* cell line (Lynch et al., 2010). More recently, Fernandes *et al.* (Fernandes et al., 2020) evaluated the application of adaptive laboratory evolution (ALE) to hypothermic culture conditions and the effect of the supplementation with productivity enhancers such as NaBu and DMSO for improved HIV-1-based VLP production in *Sf9* and *High Five* cells. Although supplementation resulted in an improvement of HIV-1- based VLP expression, the highest increase was observed by the implementation of ALE, validating the method as a compelling protocol for producing VLPs in stable insect cell lines (Fernandes et al., 2020). Puente-Massaguer et al. (Puente-Massaguer et al., 2020) assessed the transition to a novel stable cell pools (SCP) system as a potential alternative to the classic clonal cell line system to generate HIV-1 Gag-enhanced green fluorescent protein (eGFP) VLPs. SCP based systems exhibit shorter growth times and higher product yields, making them very attractive as a fast and efficient VLP production system (Puente-Massaguer et al., 2020). This approach achieved production yields like those of clonal cell lines with reduced growth times, as the adaptation phases of clonality and suspension were avoided, and stable protein production for one month (Puente-Massaguer et al., 2020). Moreover, upscaling SCP systems into bioreactor scales improved VLP production by 2-fold and reduced by-products formation (Puente-Massaguer et al., 2020). Insect cell lines thus emerge as promising expression platforms for HIV-1 VLPs, but the ability to confer the adequate glycan signature needs to be assured.

### 2.3 Immunogenicity of HIV-1-based VLPs

Natural HIV infection prompts an adaptive immune response that generates cytotoxic T lymphocytes (CTL) against Gag and Pol epitopes (Zuniga et al., 2006). Neutralizing antibodies against Env are detected several months following primoinfection. Notwithstanding, HIV is capable of bypassing this immune response given its high mutational capacity. Broad neutralizing antibodies (bNAbs) are only produced in a few infected individuals (Simek et al., 2009). The development of a protective vaccine against HIV-1 that is capable of eliciting both a high titer of bNAbs and an effective CTL response is thus required. HIV-1-based VLPs emerge as promising vaccine candidates given their ability to incorporate Env trimeric proteins on their surface, which can stimulate bNAb production, and their structure, which promotes uptake by APC and subsequently leads to a potent cellular immune response (Cervera et al., 2019).

HIV-1-based VLPs are capable of inducing potent humoral and cellular immune responses, as reported by several studies (Buonaguro et al., 2002; Buonaguro et al., 2006; Tong et al., 2014). The potent immunogenicity of HIV-1-based VLPs beings with APC uptake and consequent processing (Deml et al., 2005). Their epitopes are then presented on major histocompatibility complex (MHC) molecules and trigger immune cell activation (Deml et al., 2005). VLP-based vaccines are typically tailored to target B cells and stimulate potent antibody responses ensuing presentation by MHC class II molecules and activation of T helper cells. The VLP structure also permits cross-presentation of VLP-derived peptides on MHC class I molecules, which stimulates CTLs and thus poses an advantage for VLP-based vaccine candidates (Deml et al., 2005). Notwithstanding, co-stimulatory signals, such as cytokines, are also required for T-cell activation (Deml et al., 2005). The production of these signals can be triggered by contaminant components, such as nucleic acids and cell membranes (Deml et al., 2005). Moreover, different epitopes can be incorporated into VLPs through genetic fusion or co-expression of structural proteins (Naskalska and Pyrc, 2015). Small epitopes or complete envelope glycoproteins can be incorporated into HIV-1 Gag VLPs and consequently elicit neutralizing responses (Deml et al., 2005; Tong et al., 2014).

### 2.4 HIV-1-based VLP applications

**Figure 2:**
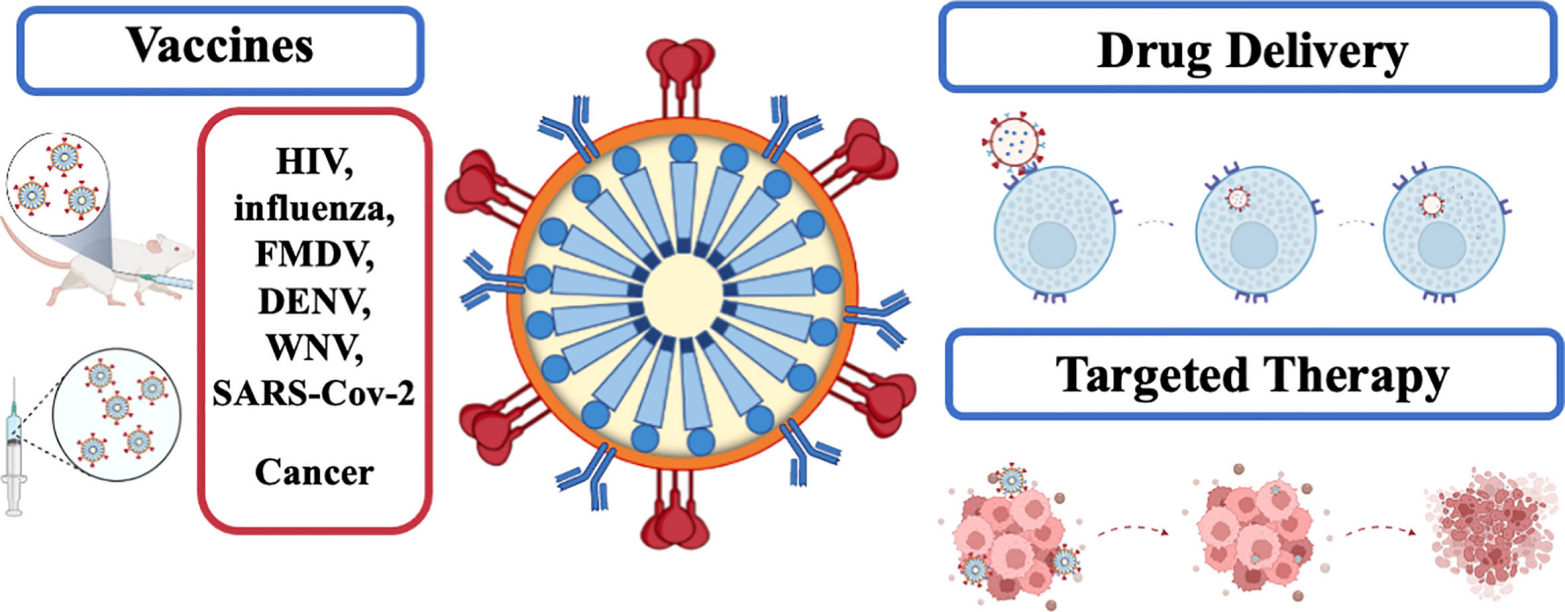
Applications of HIV1-based VLPs in medicine.

HIV-1-based VLPs are characterized by their simple self-assembly process, which requires just one polyprotein. In addition, as VLPs, they can elicit strong immune responses, encapsulate active molecules and display targeting moieties on the surface. These features make HIV-1-based VLPs attractive nanoplatforms for a wide range of medical applications, namely vaccination and drug delivery. These applications are depicted in **Figure 2**.

#### 2.4.1 HIV-1-based VLPs as vaccines

The development of therapies such as antiretrovirals (ART) and pre-exposure prophylaxis (PrEP) have reduced the mortality associated with HIV-1 infection. However, these therapies are not able to end the epidemic since ART do not eliminate the virus completely and may lose efficacy due to drug resistance, and PrEP is a preventive strategy rather than a treatment. In addition, both have long-term side effects and are not accessible yet to all countries due to relatively high cost. Therefore, the need to develop a vaccine capable of improving the construction of HIV-1 antigens has been one of the priorities in HIV-1 treatment studies (Chen et al., 2020). **Table 1** briefly describes the main vaccines developed for HIV-1 based on VLPs.

**Table 1:** VLP-based vaccines for HIV-1 (Weber et al., 1995; Tagliamonte et al., 2010; Franco et al., 2011; Ao et al., 2019; Ao et al., 2021; Beltran-Pavez et al., 2022).

Description	References
Phase I and II clinical trials: HIV-1 therapeutic p24- and p17-VLP derived from Gag capsid generated by Saccharomyces cerevisiae. The results demonstrated that it is a safe vaccine without adverse effects detected in healthy volunteers. However, impaired immune responses have been reported.	[(Weber et al., 1995)]
Pr55^Gag^-based VLP has been constructed in a baculovirus expression system and was considered an auspicious vaccine candidate.	[(Tagliamonte et al., 2010)]
The application of vaccines that enhance the immune response of cells and thus counter disease, the so-called immunotherapy, has also been the focus for HIV treatment. The CD40L, an immunostimulatory molecule, was incorporated into HIV-1 VLPs and an HIV VLP was pseudotyped with an altered Ebola virus envelope glycoprotein and both have potential to elicit a potent HIV-specific humoral immune response. A more recent study took advantage of individuals in which broadly neutralizing activity at earlier stages of HIV-1 infection had been previously detected to develop HIV-1 Gag VLPs that incorporated envelope proteins whose sequence was obtained from two of those individuals.	[(Franco et al., 2011; Ao et al., 2019; Ao et al., 2021; Beltran-Pavez et al., 2022)]

The HIV-1-based VLP vaccines can be classified into five different groups (Chen et al., 2020), depending on the construction strategy, as summarized in **Table 2**.

**Table 2:** Comparison of structural features and functional versatility of VLPs corresponding to VLP-based HIV-1 vaccine design (Rovinski et al., 1995; Wagner et al., 1996; Deml et al., 1997; Buonaguro et al., 2002; Deml et al., 2005; Tagliamonte et al., 2011; Pastori et al., 2012; Zhai et al., 2013; Chen et al., 2020).

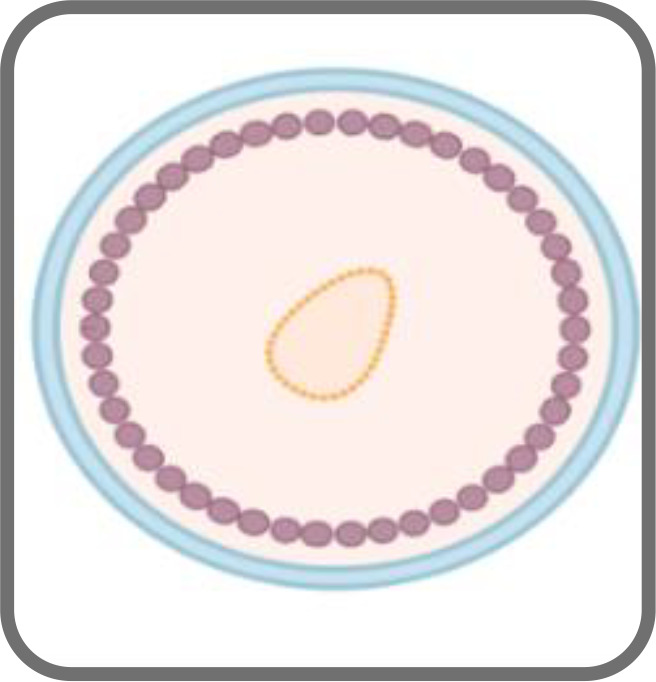	This type of design is centred on the viral capsid of native virus which is an important component that enables cell membrane assembly and budding and can enhance cellular and humoral immunity. However, it has been shown that the immunity conferred by Gag VLP varies according to structure, a fact that must be considered when developing vaccines against HIV (Chen et al., 2020).
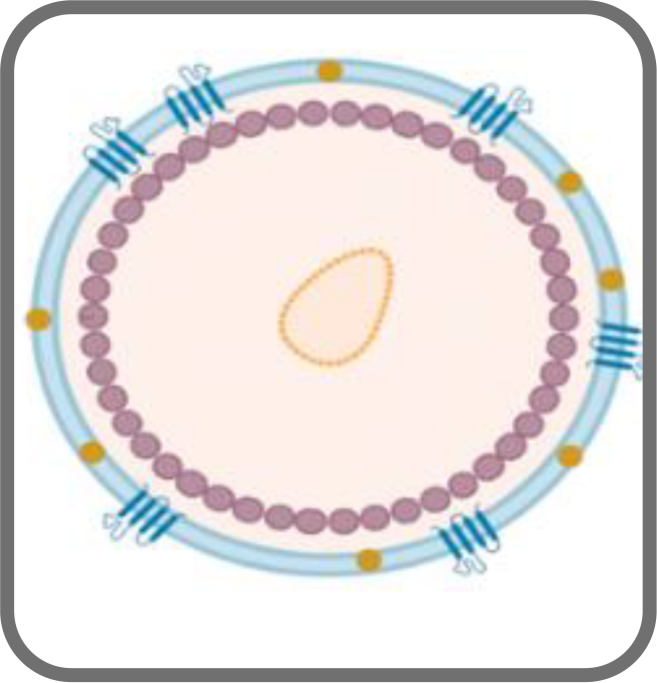	These vaccines are characterized by the presence of small antigens (epitopes) genetically fused to backbone proteins or on the surface of the VLP (Deml et al., 2005). The first strategy can generate a myriad of Gag mutants, since it is possible to either substitute commutable Gag domains with desired epitopes or fuse them to the Gag precursor (Deml et al., 2005). The existence of Env proteins on the VLP surface is particularly pertinent for robust humoral and cellular immune reactions and thus needs to be considered during vaccine design (Deml et al., 2005).
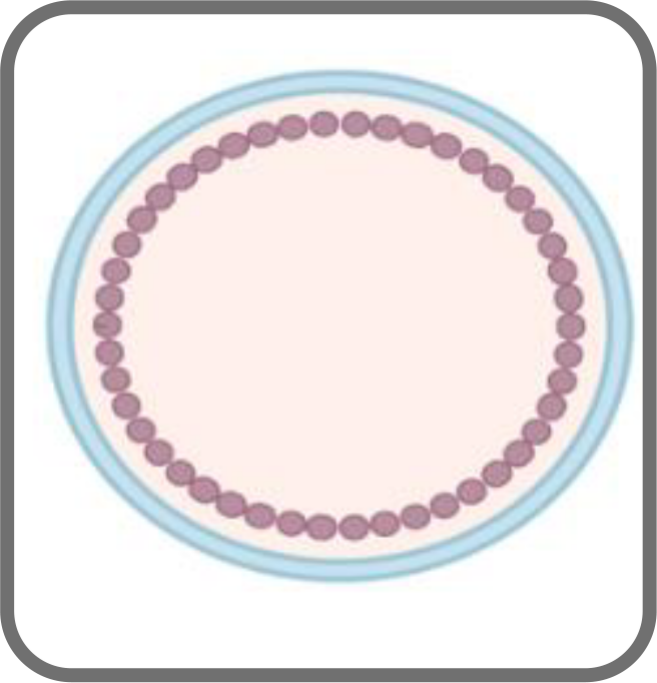	This type of vaccine relies on non-enveloped VLPs that can express HIV-1 epitopes on the surface (Chen et al., 2020). This type of vaccine depends on the architectural features of the selected epitopes (Chen et al., 2020). Nonetheless, structurally simple, non-enveloped VLPs could act as a vehicle to achieve nAbs and cytotoxic T lymphocyte (CTL) responses, as their construction is simple, and they are easily purified (Chen et al., 2020).
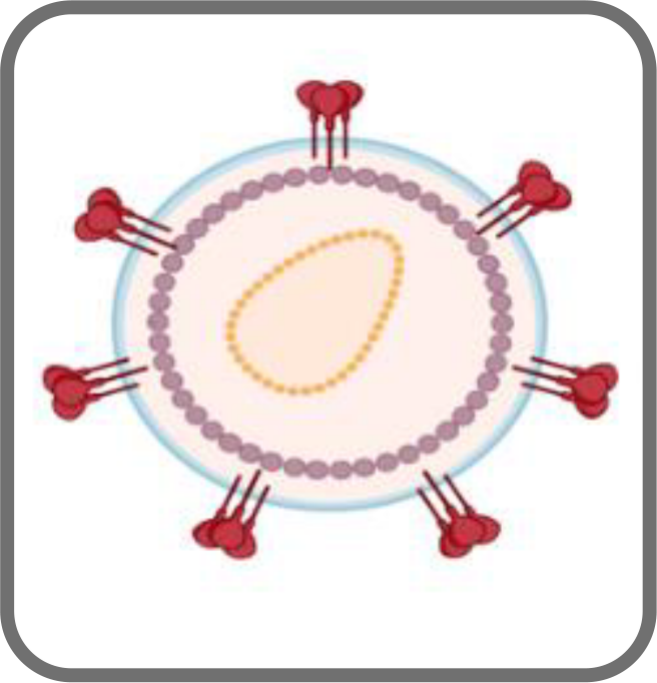	The HIV-1-based VLP vaccines have been recently constructed with “native” configurations of envelope trimers and successive envelope immunogens to trigger the production of broadly neutralizing antibodies and consequently counteract different HIV-1 strains (Chen et al., 2020). Distinct types of HIV-1 Gag VLPs have been established and evaluated in several animal models, including VLPs that express un-cleaved gp160 (Rovinski et al., 1995), monomeric gp120 (Buonaguro et al., 2002), trimeric gp140/gp41 (Tagliamonte et al., 2011), and whole Env trimer (Deml et al., 1997)
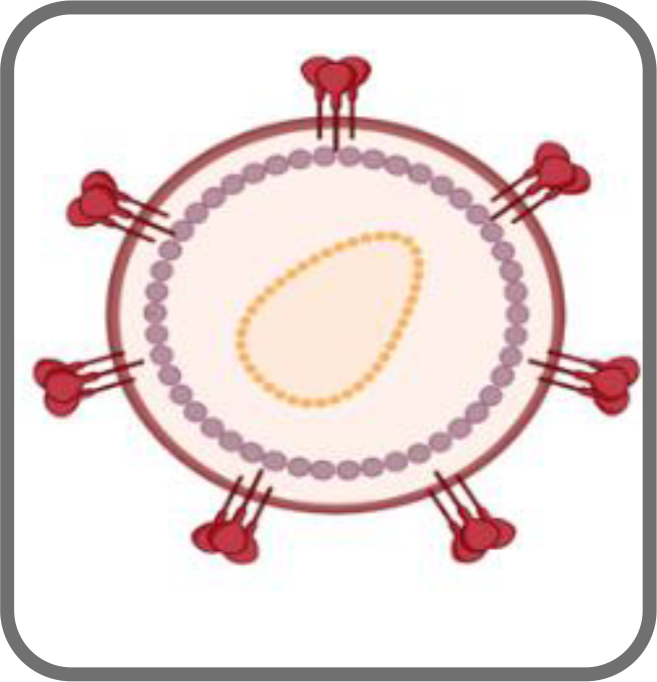	Retroviral enveloped VLPs presenting HIV-1 envelope are based on the concept that chimeric simian immunodeficiency virus (SIV) Gag VLPs exhibit altered HIV-1 Env glycoproteins with loss of glycosylation and suppression of the V1/V2 loop, which can elicit both humoral and cellular immunity and consequently neutralizing antibodies against HIV-1 infection (Chen et al., 2020). The biggest challenge in this type of HIV-1 based vaccine is focused on downstream purification (Chen et al., 2020).

HIV-1 VLPs are potential vaccine candidates for other viruses besides HIV. For instance, flaviviruses, namely dengue virus (DENV) and West Nile virus (WNV), are prevalent infectious agents in tropical and sub-tropical countries around the world transmitted mainly by mosquitoes, and effective vaccine candidates against them are required (Pierson and Diamond, 2020). The only approved vaccine against dengue is Sanofi Pasteur’s Dengvaxia^®^, which is a live attenuated vaccine composed of structural genes of four DENV strains combined with nonstructural genes of a yellow fever strain (Thomas and Yoon, 2019). The poor uptake and controversy surrounding its performance prompt the development of novel alternatives (Thomas and Yoon, 2019). HIV-1 VLPs pseudotyped with domain III (DIII) of the envelope glycoprotein of DENV and WNV were constructed in a baculovirus-insect cell host system, to develop a nanoplatform that could be used as a vaccine against DENV and WNV (Chua et al., 2013). Firstly, a chimeric receptor dubbed CD16-RIgE, composed of the ectodomain of CD16 fused to the cytoplasmic and transmembrane domains of FcεRIγ, was incorporated into Pr55Gag^HIV^ VLPs to evaluate whether the two recombinant proteins would assemble in insect cells in a similar manner as in mammalian cells (Chua et al., 2013). After confirming the successful assembly of the recombinant proteins in the VLPs, the CD16 ectodomain was replaced with the DIII from DENV and WNV, and the constructed VLPs were immunologically characterized (Chua et al., 2013). Both DIII domains were accessible on the surface of the VLPs, and the ectodomain of DIII-DENV-RIgE was shown to react against patient antibodies (Chua et al., 2013). Lastly, tests in mice revealed that a neutralization response was attained in 60% of mice (Chua et al., 2013). Further studies are required to validate this vaccine candidate, but it appears to be a promising vaccine vector.

The influenza virus originates a highly prevalent respiratory disease that provokes annual epidemics as well as sporadic pandemics (Rambaut et al., 2008). The most viable strategy to protect against influenza infection is vaccination (Steel et al., 2010). The major shortcoming of influenza vaccination is its restricted breadth of protection given the recurring replacement of prevalent strains with novel strains that are capable of evading existing antibody reactions (Steel et al., 2010). It is therefore crucial to develop novel vaccines (Steel et al., 2010). Steel et al. (Steel et al., 2010) manufactured a new immunogen composed of the conserved influenza hemagglutinin (HA) without the globular head, since this domain is thought to shield HA2, a well-conserved domain of HA that is cross-reactive between strains and possibly cross-protective (Steel et al., 2010). Headless HA molecules were incorporated into HIV-1 Gag VLPs produced in HEK-293T cells, and the resulting VLPs were combined with headless HA constructs and administered to mice. Such treatment provided protection against death and weight loss (Steel et al., 2010). The obtained results warrant further assays for the development of headless HA influenza vaccines that harness HIV-1-based VLPs as vaccine vectors (Steel et al., 2010). Another study reported the development of an influenza vaccine through the construction of HIV Gag VLPs that function as scaffolds (Venereo-Sanchez et al., 2016). A HEK293 cell clone that stably expresses HA and NA of influenza subtype H1N1 was generated, and co-expression with Gag allowed the formation of influenza Gag VLPs (Venereo-Sanchez et al., 2016). Process scalability was achieved and a yield of 138 μg of HA for 1 L of cell culture volume was attained (Venereo-Sanchez et al., 2016). Finally, mice were immunized with the developed VLPs and significant levels of HA-specific IgA, Ig1 and Ig2 were observed, together with robust protection following a lethal intranasal challenge, revealing the potential of this influenza vaccine (Venereo-Sanchez et al., 2016).

Foot and Mouth Disease (FMD), an infection that affects livestock, is caused by Foot-and-Mouth-Disease Virus (FMDV) and is mostly controlled through vaccination (Grubman and Baxt, 2004). The vaccines that are currently in use are based on inactivated FMDV in combination with different adjuvants (Belsham, 2020). Albeit effective, these vaccines display several shortcomings, namely transient protection, short shelf life and requirement of multiple doses (Belsham, 2020). A novel FMD vaccine candidate was produced through co-expression of a fusion construct comprised of the rabies virus glycoprotein and the FMDV G-H loop, and the HIV-1 Gag polyprotein (Fontana et al., 2021). The *gag* gene was fused to the gene that encodes GFP for quantification and characterization, and chemical additives were added to optimize VLP production (Fontana et al., 2021). Lastly, the ability of the constructed VLPs to elicit a specific immune response in mice was evaluated, and antibodies against the rabies virus glycoprotein and against the FMDV G-H loop (Fontana et al., 2021). It was thus possible to attain the proof-of-concept of this nanoplatform as a FMDV vaccine candidate, and additional studies are necessary to determine the extent of the protection conferred by these VLPs (Fontana et al., 2021).

The most recent pandemic of COVID-19 caused by severe acute respiratory syndrome coronavirus 2 (SARS-CoV-2) has had nefarious effects in public health and economy worldwide (Hu et al., 2021). This outbreak has prompted the swift development of vaccine candidates, some of which were approved for use (Forni et al., 2021). As of June 7, 2022, over 11 billion doses have been administered (World Health, 2022). The COVID-19 vaccines that have been approved thus far rely on mRNA technology, adenovirus vector systems or recombinant technology (Heath et al., 2021; Teijaro and Farber, 2021). mRNA vaccines encode for the SARS-Cov-2 spike (S) protein, rely on lipid nanoparticles as carriers and are taken up by DCs (Teijaro and Farber, 2021). Adenovirus-based vaccines harness non-replicating adenovirus vectors for the delivery of DNA encoding the S protein to DCs (Teijaro and Farber, 2021). Lastly, recombinant vaccines are based on a recombinant nanoparticle S protein (Heath et al., 2021). VLPs could also be explored as COVID-19 vaccine candidates, given their intrinsic characteristics (Benjamin et al., 2020). HIV-1 Gag VLPs display a similar size to WT SARS-CoV-2 viral particles and can therefore be used as platforms for the display of its epitopes (Yao et al., 2020; Lavado-García et al., 2021). HEK293 cells were thus utilized to produce chimeric VLPs containing the HIV-1 Gag polyprotein and the S protein (Boix-Besora et al., 2022). These VLPs were characterized and purified, and the production was optimized (Boix-Besora et al., 2022). Furthermore, it was possible to produce them at a bioreactor scale, achieving concentrations of around 3.5 x 10^9^ VLPs/ml (Boix-Besora et al., 2022). Finally, the constructed VLPs were submitted to downstream processing for purification, achieving a purity of 31.1% (Boix-Besora et al., 2022). This approach served as a kick-off study to produce HIV-1-based VLPs as COVID-19 vaccine candidates, although further optimization and immunological assays will be required to support their full potential.

##### 2.4.1.1 HIV-1-Based VLPs as therapeutic cancer vaccines

The uptake of VLPs occurs in several important immune cells, namely dendritic cells, macrophages, mast cells and B cells. The innate binding capability to their natural target cells is frequently lost following modification. This loss allows entry into the endogenous intracellular APCs and leads to the activation of CTLs. Such mechanism is called cross-priming and improves immune defense against viruses and tumors. Therefore, VLPs have been modified with the aim of presenting tumor-associated antigens (Tagliamonte et al., 2010).

Di Bonito et al. (Di Bonito et al., 2009) studied the incorporation of a mutant of HIV-1 Nef (Nef^mut^) into HIV-1 based VLPs with the aim of triggering a CD8+ T cell immune response, reported the cross-presentation of VLP-associated Nef^mut^ in APCs and concluded that Nef^mut^ VLPs efficiently penetrate B-Lymphoblastoid cell lines (B-LCLs). The most substantial benefit of utilizing the Nef^mut^ VLP platform over other approaches is that Nef^mut^ VLPs, apart from the Nef^mut^-based fusion protein, are comprised of only four viral components. Furthermore, distinct receptors can be used to pseudotype Nef^mut^ VLPs, which can in turn overcome the antibody-dependent neutralization that occurs following numerous immunizations.

Lambricht et al. (Lambricht et al., 2016) hypothesize that the *in vivo* electroporation of plasmids encoding HIV-1 Gag (pGag) could enhance immune responses. Firstly, the formation of particles following cell transfection with pGag was determined. Then, the induction of innate immunity was assessed *in vivo*. Lastly, the role of VLP assembly on the pGag immunomodulatory effects *in vivo* was evaluated. The obtained results show that the assembly of HIV-1 Gag VLPs does not influence the antigen-specific immunogenicity *in vivo*. On the other hand, electroporation of pGag or pGag* (pGag mutated version) alone delayed tumor growth concomitantly with therapeutic immunizations, which proves the Gag encoding plasmid can spur the innate immune response. Additionally, the HIV-1 Gag VLPs efficiently stimulate dendritic cell maturation, which are innate immune cells that penetrate tumors and are critical in the development of an anti-tumor T cell immunity (Wylie et al., 2019) by augmenting the expression of MHC-I and II and costimulatory molecules like CD80 and CD86 (Lambricht et al., 2016).

Buonaguro *et al.* conducted several studies demonstrating that Pr55gag VLPs stimulate myeloid-derived dendritic cells (MDDCs) cultured *in vitro via* TLR-3 and -9 signaling (Buonaguro et al., 2006; Buonaguro et al., 2009). It has also been observed that HIV-1 Gag VLPs can activate natural killer cells in mice, which are particularly relevant in the antitumor response (Chang et al., 2012; Lambricht et al., 2016). Taken together, the results obtained from these studies support the rationale that HIV-1-based VLPs can act as promising anti-tumor vaccines, even though it is still a rather unexplored field.

#### 2.4.2 HIV-1-based VLPs as delivery vehicles

**Figure 3:**
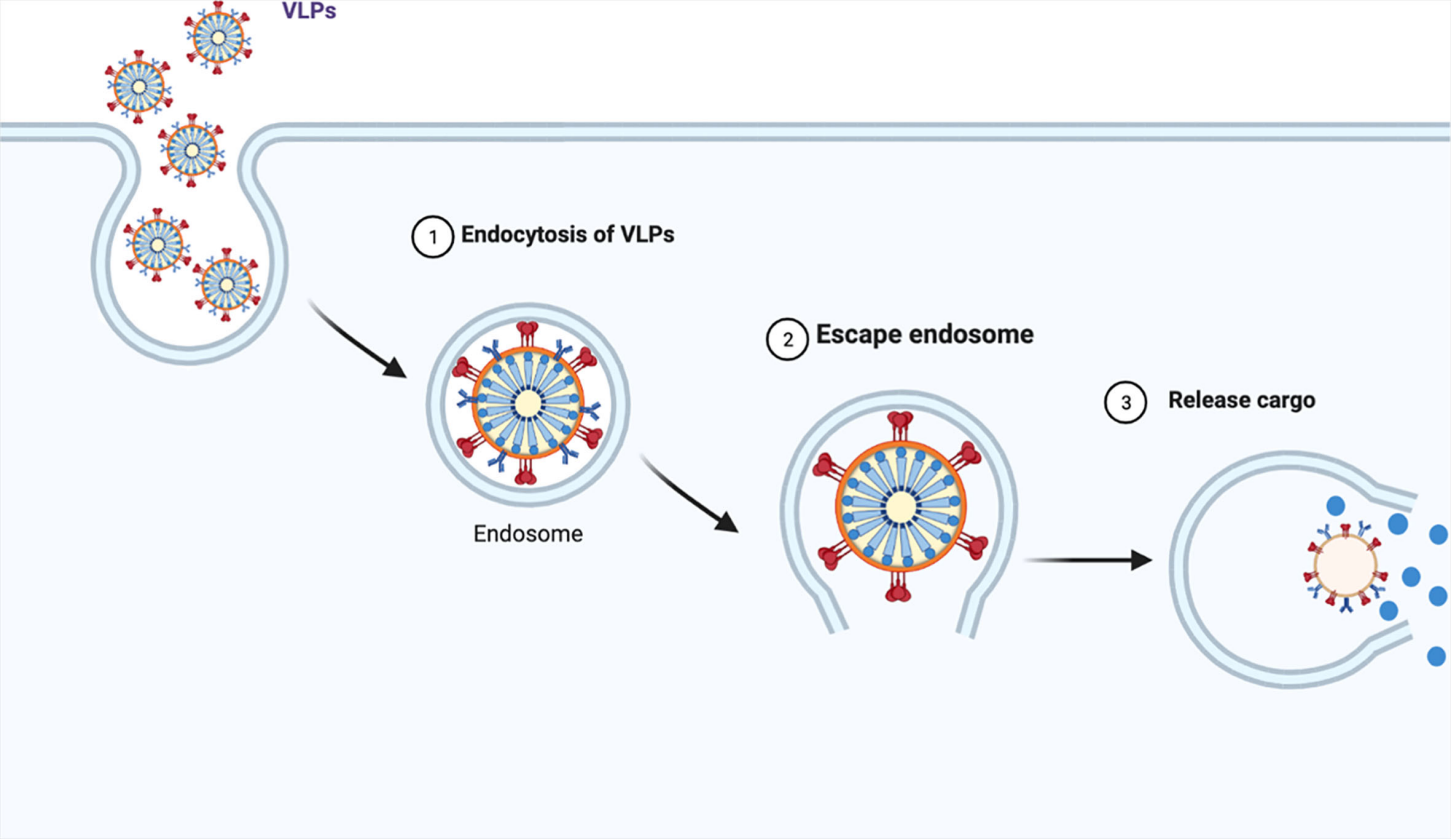
VLPs undergo endocytosis and following intracellular release of the vesicles, they migrate along the cytoskeleton to merge with early endosomes (1) (Zdanowicz and Chroboczek, 2016). Once endocytosed, the VLPs escape the endosome and then disassemble to release their cargo (2 and 3).

VLPs can bear targeting peptides, proteins and other active molecules on the surface as well as enclose such moieties on the interior (Zdanowicz and Chroboczek, 2016). This enables them to act as delivery vehicles to target specific cells, tissues or organs, releasing the cargo inside the cells (Zdanowicz and Chroboczek, 2016). **Figure 3** illustrates the mechanism of endocytosis of the VLPs in cells.

Drug delivery is normally hampered because synthetic NPs undergo this route (Zdanowicz and Chroboczek, 2016). Indeed, drug delivery is the primary shortcoming of most modern therapies (Zdanowicz and Chroboczek, 2016). VLP specificity can be acquired by the ability of the native virus to target and infiltrate different cell types by means of the surface receptors (Naskalska and Pyrc, 2015). This ability reduces side and off-target effects, protecting drug stability and activity making these nanoplatforms highly desirable for therapeutic carriers (Zdanowicz and Chroboczek, 2016).

Although nucleic acids are their natural cargo, VLPs are adaptable platforms that can be altered to incorporate and deliver a broad variety of small or large molecules, with both biologic and synthetic origin (Chung et al., 2020). The self-assembly process of viruses to package their genome inside the capsid can be employed to load functional cargos within the VLPs (Chung et al., 2020). Typically, *in vitro* conditions such as pH, buffer conditions and ionic strength can trigger capsid disassembly and consequent release of viral genetic material (Chung et al., 2020; Alvandi et al., 2022). Both electrostatic and binding interactions need to be considered during self-assembly (Chung et al., 2020; Alvandi et al., 2022). Besides self-assembly, encapsulation can also be achieved by infusion, which relies on the existing pores on the VLPs surface and that allow the dispersion of small molecules into and out of the capsid (Chung et al., 2020; Alvandi et al., 2022). Genetic engineering can also be performed to alter the internal cavity of the VLPs for packaging (Ma et al., 2012).

Regarding HIV VLPs, one study took advantage of the abundance of the Gag polyprotein within the virion to fuse it with different proteins of interest (Robert et al., 2017). 293SF-pacLV cells were stimulated with cumate and doxycycline for VLP assembly and for expression of the vesicular stomatitis virus glycoprotein (VSVG), the latter being involved in VLP binding to the target cells (Robert et al., 2017). A cytomegalovirus (CMV) cassette containing the *gag-pol* gene together with a Rev Responsive Element (RRE) was also introduced into the 293SF-pacLV cells (Robert et al., 2017). The expression of Rev is also triggered following induction with cumate and doxycycline, and Rev can then bind to the RRE, forming a complex (Robert et al., 2017). The nuclear export signal present on Rev recruits Crm1 and Ran-GTP, facilitating the nuclear export of the Gag-Pol transcript, and consequently permitting the translation of Gag and the HIV-1 protease (Robert et al., 2017) (Booth et al., 2014). It was thus possible to produce VLPs containing native HIV-1 Gag (Robert et al., 2017). This study reported the efficient delivery of green fluorescent protein (GFP), a synthetic transcription factor (the cumate transactivator of the inducible CR5 promoter) and a natural transcription factor (the human Krüppel-like factor 4) to human cell lines and the possibility to utilize protein engineering to alter their localization within the cells (Robert et al., 2017). This approach could be tackled for the incorporation of recombinant proteins into VLPs and thus develop novel platforms that can be employed in the clinical field (Robert et al., 2017). For instance, enzymes such as Cas9 could be packaged into VLPs for genome editing purposes. This was achieved by Hamilton et al. (Hamilton et al., 2021). A plasmid combining *Streptococcus pyogenes* Cas9 with the C terminus of the Gag polyprotein and a plasmid containing the expression cassettes for both the mNeonGreen protein and a single guide RNA (sgRNA) were constructed (Hamilton et al., 2021). A linker that can be cleaved by the HIV-1 protease was placed between Gag and Cas9 to prompt the separation of Cas9 from Gag during VLP assembly (Hamilton et al., 2021). The Cas9-VLPs were pseudotyped with VSVG and different plasmid ratios were tested for optimization (Hamilton et al., 2021). A sgRNA aimed at the β-2 microglobulin (B2M) gene was used to assess the gene editing activity, and both genetic knockout and loss of protein expression were observed (Hamilton et al., 2021). It was also possible to edit primary human T cells by treating them with distinct Cas9-VLPs targeting *B2M* and *TRAC* (Hamilton et al., 2021). Lastly, the HIV-1 Env protein was harnessed to achieve CD4^+^ T cell tropism (Hamilton et al., 2021). Other strategies have also been tested for the packaging of Cas9 ribonucleoproteins into HIV-1-derived VLPs, including the combination of Cas9 with the accessory protein Vpr, which proved to be efficient at promoting gene editing in immortalized cell lines (Indikova and Indik, 2020), and the fusion of aptamer binding proteins to the C terminus of the Gag polyprotein following the placement of RNA aptamers in the sgRNA tetraloop, which displayed an efficient gene editing activity and on-target to off-target discrimination (Lyu et al., 2019). VLPs may thus be a relevant tool to achieve targeted delivery of gene editing nucleases. The main limitations encountered during these studies include the difficulty of producing VLPs with specific proteins packaged in suspension without requiring serum and the inefficient processing of the Gag chimeric proteins (Robert et al., 2017; Hamilton et al., 2021). Further studies are thus required to optimize VLP production and improve delivery, with the prospect of having VLPs as delivery vehicles soon.

Besides trapping molecules inside VLPs, vectorization of VLPs can also be done with different molecules to attain the desired targeting properties. The structure of the viruses utilized in VLP assembly is well characterized, allowing for the selection of sequences amenable to genetic modifications and localization of N- and C-termini of their components (Zdanowicz and Chroboczek, 2016). The insertion of peptides (up to 50 amino acids) or small proteins, which have reached a relevant role as targeting moieties, can then be conducted through protein fusion on the surface of VLPs (Zdanowicz and Chroboczek, 2016). Although peptides larger than 30 residues can compromise VLP capsid assembly, many smaller peptides have proven to possess excellent vector characteristics (Zdanowicz and Chroboczek, 2016). For larger peptides, functionalization of the VLP surface can be achieved by direct conjugation to accessible lysine or cysteine residues (Shirbaghaee and Bolhassani, 2016).

The Gag core of the HIV-1 based VLP is a flexible and adaptable system with a lipid envelope that can be altered to include various types of proteins or antigens (Lavado-García et al., 2021). Combining standard conjugation strategies with budding peptide sequence vectors, the outer surface of virus particles capsids can be functionalized to increase specificity and affinity to target cells (Ma et al., 2012; Van Kan-Davelaar et al., 2014; Shirbaghaee and Bolhassani, 2016; Zdanowicz and Chroboczek, 2016). HIV-1 TAT’s positively charged residues (Arg, Lys) react with the negatively charged membrane promoting membrane permeation. When combined with nanocarriers, it creates a vessel with enhanced intracellular delivery properties of the cargo drug (Torchilin et al., 2001; Wei et al., 2009; Trabulo et al., 2010; Kaczmarczyk et al., 2011; Somiya et al., 2017).

Functionalization for targeting in HIV VLPs has been mostly reported in potential vaccines against HIV infection (Ao et al., 2019; Barnowski et al., 2019; Ao et al., 2021). The FMDV vaccine candidate (Fontana et al., 2021) and the most recently developed SARS-CoV-2 HIV VLP-based vaccine (Boix-Besora et al., 2022) also considered the functionalization to enhance their yields. Another study reported the introduction of an altered form of flagellin that does not trigger strong immune responses as a booster into HIV-based particles aimed at B-cells, through expression of a plasmid encoding a membrane-anchored form of flagellin (Barnowski et al., 2019)

Concerning targeted delivery, our group combined an *in silico* study with an experimental approach aimed at the development of a targeting motif that associates the single-chain variable domain fragment (scFv) of trastuzumab, a monoclonal antibody against the human epidermal growth factor receptor type 2 (HER2), with the HIV protein gp41 (Santos et al., 2021). Firstly, computational tools were used to perform docking studies to ascertain which residue in gp41 was the most adequate to fuse with the scFv. This permitted the construction of a plasmid containing the protein sequence scFv-HER2_gp41 for mammalian expression (Santos et al., 2021). The HEK-293T cell line was used to perform transient transfection with the constructed vector, and the Western Blot technique was employed to assess the expression of the plasmid in the cells (Santos et al., 2021). It was possible to validate the formation of the scFv-HER2_gp41 motif, which is the first successful step towards constructing HIV-1-based VLPs that express the scFv and ultimately developing a novel tool for targeted therapy.

While the use of chimeric VLPs with targeting moieties is still in early stages of development, the ever-growing libraries of available viruses, small peptides, and molecules with high targeting specificity for designed targets will promote new trends in this field. Indeed, the use of VLPs as diagnostic, preventive and therapeutic platforms for various diseases will contribute to the improvement of already defined therapies or even advances in the field of theranostics (Yildiz et al., 2011; Van Kan-Davelaar et al., 2014; Shirbaghaee and Bolhassani, 2016; Zdanowicz and Chroboczek, 2016). It is, however, important to consider that VLPs possess features that are deleterious for their use as drug delivery vehicles (Zdanowicz and Chroboczek, 2016). One of these features is the ability to induce an immune response by proteinaceous particle application (Zdanowicz and Chroboczek, 2016). Strong cellular and humoral immune reactions are elicited by the repetitive, dense and organized architecture of VLPs (Zdanowicz and Chroboczek, 2016). Furthermore, the uptake of VLPs by dendritic cells and their transport to lymph nodes prompts a T-cell response which is crucial in cell-mediated immunity (Zdanowicz and Chroboczek, 2016). This indicates that the immune response may hamper VLP use for drug delivery (Zdanowicz and Chroboczek, 2016). Nonetheless, the benefits of using VLPs as drug delivery vectors may outweigh the possible disadvantages, deeming further studies in this field necessary.

## 3 Conclusions and future perspectives

The fields of nanotechnology and protein engineering have significantly evolved in recent years, allowing for the development of vaccine carriers, drug delivery vehicles and bioimaging tools (Mitchell et al., 2021). VLPs emerged as relevant platforms useful for a broad range of biomedical applications given their properties, which include the fact that they are non-infectious nanostructures that maintain some of the characteristics of the viruses they originate from (e.g., composition, antigenicity, and specific targeting) (Nooraei et al., 2021). These properties make them an appealing alternative to other synthetic NP systems (Nooraei et al., 2021). The ability to design VLPs to carry antigenic structures has enabled their use as vaccination agents (Peabody et al., 2008; Francica et al., 2021). Apart from vaccines, VLPs may have other biomedical applications, as shown by several recent studies (Li et al., 2009; Ashley et al., 2011; Pokorski et al., 2011). Owing to their biocompatibility, stability, and structure, VLPs can be engineered to encapsulate proteins, peptides, nucleic acids, imaging agents, drugs, quantum dots or other types of molecules (Nooraei et al., 2021). They can also display targeting moieties on the outer surface *via* chemical and genetic modifications, allowing for a tunable specificity (Bustos-Jaimes et al., 2017; Zackova Suchanova et al., 2017). However, modifications on the VLP surface or in the capsid subunits structure can prevent VLP self-assembly or interfere with its structure and consequently alter its behavior and biological characteristics (Grgacic and Anderson, 2006). Moreover, due to their structure, VLPs may elicit a strong immune response, which is relevant for vaccination but may hinder their use as drug delivery vehicles and/or bioimaging tools (Donaldson et al., 2018). The favorable properties displayed by VLPs might outweigh the possible disadvantages, prompting further studies to modify VLPs, optimize their production and assess their effects both *in vitro* and *in vivo*, with the prospect of implementing their use in the clinical field.

HIV-1-based-VLPs are relevant nanoplatforms due to their simple self-assembly process, which only requires the Gag polyprotein, their enveloped surface that facilitates functionalization, and their ability to elicit innate and adaptive immune responses (Cervera et al., 2013; Gonelli et al., 2019; Boix-Besora et al., 2022). Brought together these characteristics turn HIV-1-based-VLPs into highly versatile and unique platforms for vaccination and drug delivery. These VLPs can be explored as vaccine candidates for HIV, but also for other viruses, such as DENV, WNV, influenza, FMDV and SARS-CoV-2 (Steel et al., 2010; Chua et al., 2013; Venereo-Sanchez et al., 2016; Fontana et al., 2021; Boix-Besora et al., 2022). Regarding drug delivery, the inner core is large enough to carry other molecules (e.g., small molecules or proteins) or nanoparticles, protecting them from the biological environment (Hamilton et al., 2021). In addition, HIV-1-based VLPs can be functionalized on the surface to achieve target specificity (Santos et al., 2021). Notwithstanding, HIV-1-based VLPs also display several shortcomings that are particularly relevant for their usage in the clinical setting. Regarding vaccination, the formation of immature virions may be insufficient to elicit adequate immune responses, whereas mature virions may incorporate their genetic material into the host genome, which may have nefarious effects (Alvarez-Fernandez et al., 2012; Chojnacki et al., 2012). Moreover, the production process entails contamination with cellular components, namely exosomes, microvesicles and RNA, the latter functioning as a scaffold for Gag VLP assembly, which is a major handicap for the medical applicability of HIV-1-based VLPs (Cimarelli et al., 2000; Steppert et al., 2017). Lastly, the adequate expression systems must be carefully chosen given the necessity of an adequate glycosylation profile for VLP stability, solubility, safety and efficacy (Gupta and Shukla, 2018). These are the major constraints to the implementation of HIV-1-based VLPs in the clinical setting that must be cautiously considered during the design of HIV-1-based VLP studies. Although these shortcomings seem to discourage the investment in HIV-1-based VLPs for medical purposes, promising results have been achieved (Santos et al., 2021; Beltran-Pavez et al., 2022; Boix-Besora et al., 2022). Moreover, the recent advances in the fields of protein engineering and genetic modification can be exploited to circumvent these difficulties and consequently produce HIV-1-based VLPs devoid of most hindrances. Their potential has been mainly explored for the development vaccine candidates, but their resourcefulness combined with specific targeting molecules may allow the development of novel original nanoplatforms useful as drug delivery agents to tackle unmet clinical needs.

## Author contributions

SM, RS, CR, and JS wrote the main manuscript. RM prepared **Figures 1** and **2**. JC and SCV were responsible for review, editing, and funding. RM was responsible for conceptualization, supervision, review, funding, and project administration. All authors contributed to the article and approved the submitted version.

## Funding

This work was supported by the FCT through projects UID/Multi/04349/2020, PTDC/QUI-NUC/30147/2017, PTDC/QUI-OUT/32243/2017 and PTDC/QUI-OUT/3854/2021.

## Acknowledgments

RM, SCV, and JC gratefully acknowledge support from the FCT through projects UID/Multi/04349/2020, PTDC/QUINUC/30147/2017, PTDC/QUI-OUT/32243/2017 and PTDC/QUI-OUT/3854/2021. RS thanks FCT for a PhD scholarship (DFA/BD/4978/2020).

## Conflict of interest

The authors declare that the research was conducted in the absence of any commercial or financial relationships that could be construed as a potential conflict of interest.

## Publisher’s note

All claims expressed in this article are solely those of the authors and do not necessarily represent those of their affiliated organizations, or those of the publisher, the editors and the reviewers. Any product that may be evaluated in this article, or claim that may be made by its manufacturer, is not guaranteed or endorsed by the publisher.
